# A Phase 1/2 Randomized Study to Evaluate the Safety, Tolerability, and Immunogenicity of Nucleoside-Modified Messenger RNA Influenza Vaccines in Healthy Adults

**DOI:** 10.3390/vaccines13040383

**Published:** 2025-04-03

**Authors:** Angela Branche, Mark J. Mulligan, Alok Maniar, Orlando Puente, Islamiat Oladipupo, Graham Crowther, Agnieszka M. Zareba, Zhuobiao Yi, Ingrid Scully, Emily Gomme, Kenneth Koury, Nicholas Kitchin, Pirada Suphaphiphat Allen, Annaliesa S. Anderson, Alejandra Gurtman, Kelly Lindert

**Affiliations:** 1 Department of Medicine, Division of Infectious Diseases, University of Rochester, Rochester, NY 14642, USA; 2 New York University (NYU) Vaccine Center, NYU Grossman School of Medicine, New York, NY 10016, USA; 3 Vaccine Research and Development, Pfizer Inc., Pearl River, NY 10965, USA; 4 Miami Dade Medical Research Institute, Miami, FL 33176, USA; 5 Vaccine Research and Development, Pfizer Ltd., Hurley SL6 6RJ, UK; 6 Vaccine Research and Development, Pfizer Inc., Collegeville, PA 19426, USA; 7 Vaccine Research and Development, Pfizer Inc., Cambridge, MA 02139, USA

**Keywords:** influenza, vaccination, modRNA, immunogenicity, safety, tolerability

## Abstract

**Background/Objectives:** Circulating influenza strains antigenically differing from vaccine antigens increase disease burden by decreasing vaccine efficacy. Nucleoside-modified mRNA (modRNA) influenza vaccines may facilitate rapid production allowing later antigen selection and improved antigenic similarity compared to circulating strains. We studied different influenza modRNA vaccine (IRV) formulations and dose levels. **Methods:** This phase 1/2 randomized study evaluated IRV safety/tolerability and immunogenicity in healthy 18- through 85-year-olds. Based on safety and immunogenicity for different IRV doses, schedules, and valencies versus the quadrivalent influenza vaccine (QIV; Fluzone High-Dose Quadrivalent, Sanofi Pasteur) in phase 1 (65–85-year-olds), quadrivalent IRV (qIRV) was further evaluated in 65- through 85-year-olds and 18- through 64-year-olds in phase 2, leading to phase 3 dose selection. **Results:** Phase 1 (65–85-year-olds) safety/tolerability and immunogenicity findings supported qIRV 30-µg and 60-µg phase 2 assessment (18–85-year-olds, N = 610). qIRV was well tolerated. Injection site pain was the most frequently reported local reaction. Reactogenicity event incidences ≤ 7 days postvaccination for qIRV were generally higher versus QIV, observed more frequently in 18- through 64-year-olds than 65- through 85-year-olds, and showed dose-related trends (60 μg > 30 μg). qIRV and QIV adverse event profiles in 65- through 85-year-olds were similar. There were higher postvaccination hemagglutination inhibition assay geometric mean titers and fold rises and seroconversion rates observed with qIRV versus QIV for A strains, with no consistent pattern for B strains. Cell-mediated immune responses to qIRV by Day 7 showed overall higher T-cell responses against all strains versus QIV. Antibody and cell-mediated immune responses showed comparable trends across qIRV doses in 18- through 85-year-olds; a dose-related pattern was observed in 65- through 85-year-olds (60 μg > 30 μg). **Conclusions:** Phase 3 investigations of qIRV 60 µg in older adults and qIRV 30 µg in younger adults are warranted (ClinicalTrials.gov Identifier: NCT05052697).

## 1. Introduction

Influenza is a major cause of morbidity and mortality worldwide [1]. Influenza infection can cause febrile illness with respiratory and systemic symptoms [2]. The risks of complications and hospitalization are higher in individuals of any age with certain underlying medical conditions, as well as in those ≥ 65 years of age and young children [3]. In the United States, the in-season estimates of influenza-related burden for the 2023/2024 influenza season were approximately 34 to 75 million cases of influenza, resulting in 15 to 33 million medical visits, 380,000 to 900,000 hospitalizations, and 17,000 to 100,000 deaths [4]. These estimates indicate that influenza remains a major public health challenge.

In the United States, multivalent inactivated vaccines targeting three (A/H1N1, A/H3N2, and B/Victoria) or four (those same strains plus B/Yamagata) influenza strains are produced for routine seasonal immunization against influenza [5]. Viruses forming the basis of inactivated vaccines are replicated either in embryonated hens’ eggs or mammalian cell lines [6]. Because of the continual variability of circulating influenza viruses resulting from antigenic drift, ongoing global influenza virus surveillance in both hemispheres is required to identify which strains should be targeted by seasonal influenza vaccines [7].

Accurate prediction of emerging influenza strains remains complex. Manufacturing of inactivated influenza vaccines typically starts 6 to 8 months before the beginning of the influenza season, during which time substantial further antigenic drift can occur [7,8]. This can lead to differences in antigenicity between the current circulating strain(s) and the vaccine and, thus, reduced vaccine effectiveness, which can also be decreased by virus egg adaptive mutations [8,9]. When the vaccine and circulating strains are antigenically similar, vaccine efficacy in the overall population is approximately 40% to 60%; however, vaccine efficacy as low as 19% has been observed in the past decade, in part because of the antigenic drift in circulating viruses occurring after strain selection (for example, the antigenic drift in circulating A/H3N2 viruses that were observed in the 2014/2015 season) [10].

The development of nucleoside-modified messenger RNA (modRNA) vaccines has facilitated the rapid, scalable production of seasonal vaccines [11]. The modRNA platform has enabled acceleration of manufacturing processes, which may allow decisions on influenza strain selection to be made later in the year than with inactivated vaccines, and reduces the chances of antigenic dissimilarity between selected vaccine antigens and circulating strains [12]. The platform can produce large numbers of doses for administration and can induce a multifaceted immune response, including both humoral and cell-mediated responses [13]. Of note, two safe and efficacious lipid nanoparticle-encapsulated modRNA vaccines encoding the SARS-CoV-2 spike protein were developed and successfully deployed in response to the global public health emergency presented by the COVID-19 pandemic [14,15].

Preclinical studies have demonstrated the ability of influenza modRNA vaccines (IRVs) to induce robust humoral and cellular responses with a favorable tolerability profile [16]. Additionally, phase 1/2 clinical trials of an IRV have shown robust immunogenicity with no safety concerns [17,18]. To clinically assess the modRNA platform for influenza vaccines and to support late-stage clinical trial development, we report safety and immunogenicity results from a phase 1/2 study of different formulations and dose levels of an IRV.

## 2. Materials and Methods

### 2.1. Study Design and Participants

This phase 1/2, randomized, single-blind study evaluated the safety, tolerability, and immunogenicity of a modRNA vaccine against influenza in healthy adults from the United States (ClinicalTrials.gov Identifier: NCT05052697). The investigational plan included two substudies. Substudy A (phase 1; conducted from 13 September 2021 to 4 August 2022) assessed modRNA influenza vaccines of different valencies and dose levels, and substudy B (phase 1/2; conducted from 14 February 2022 to 27 January 2023) used initial enrollment and expanded enrollment stages to determine the vaccine and dose level for the phase 3 investigation.

Substudy A enrolled healthy 65- through 85-year-olds and included those with a preexisting, stable disease (i.e., disease not requiring substantial change in therapy or hospitalization for worsening disease during the 6 weeks before enrollment). Individuals were excluded if they had received an influenza vaccine within 6 months before study vaccination. Substudy B enrolled healthy 18- through 85-year-olds; those 65 through 85 years old were required to have received a licensed influenza vaccination for the preceding 2021/2022 Northern Hemisphere influenza season more than 4 months before the study vaccination but were excluded if they had received a licensed influenza vaccination for the 2022/2023 season. Exclusion criteria common to both substudies were known or suspected immunodeficiency, history of severe adverse reaction associated with a vaccine and/or severe allergic reaction (e.g., anaphylaxis) to any component of the study vaccines, allergy to egg or chicken proteins, substantial exposure to SARS-CoV-2 or influenza within the last 14 days, and individuals planning to receive a modRNA vaccine against COVID-19 within 60 days (substudy A) or 28 days (substudy B) of study vaccination. Full eligibility criteria for both substudies are provided in the Supplementary Materials.

The study was conducted in accordance with the protocol, consensus ethical principles derived from international guidelines including the Declaration of Helsinki, the Council for International Organizations of Medical Sciences International Ethical Guidelines, applicable International Conference on Harmonisation Good Clinical Practice guidelines, and other applicable laws and regulations, including privacy laws. Participants provided written informed consent before enrollment.

### 2.2. Study Intervention

The IRVs used in this study encoded hemagglutinins for seasonally adapted influenza strains. In substudy A, participants were randomized to receive either monovalent IRV (mIRV; 3.75 to 30 µg dose level encoding either one A or B strain), bivalent IRV (bIRV; 15 to 30 µg dose level encoding various combinations of A and B strains), or quadrivalent IRV (qIRV; 30 µg dose level encoding two A and two B strains; see Supplementary Materials for further details on the design and study vaccines in substudy A). Safety and immunogenicity data up to at least 1 week after the last vaccination from substudy A and subsequently a phase 1 initial enrollment stage in substudy B were reviewed by an independent review committee to establish vaccine valency and dosing for phase 2. If deemed acceptable, an expanded enrollment stage in substudy B was then conducted in 65- through 85-year-old and 18- through 64-year-old participants. Results for substudy A and for the phase 2 expanded enrollment stage of substudy B for dose groups selected for further evaluation in phase 3 studies are presented here. This included adults 65 through 85 years of age who received a single dose of either qIRV 30 µg or qIRV 60 µg or a licensed quadrivalent influenza vaccine (QIV; control; Fluzone High-Dose Quadrivalent, Sanofi Pasteur), and adults 18 through 64 years of age who received a single dose of either qIRV 30 µg or qIRV 60 µg.

Strain composition for vaccines included in substudy A and substudy B are summarized in Table S1. All vaccines were administered intramuscularly into the deltoid muscle.

### 2.3. Objectives and Endpoints

#### 2.3.1. Safety

The primary objective was to describe the safety and tolerability of IRV formulations. Safety endpoints included the percentages of participants reporting solicited local reactions and solicited systemic events within 7 days after each vaccination overall and by severity (Table S2); unsolicited adverse events (AEs) through 1 month after the last vaccination; and unsolicited serious AEs (SAEs) through 6 months after the last vaccination. In addition, AEs of special interest (AESIs) including diagnoses of myocarditis or pericarditis and influenza were monitored through 4 weeks and 6 months after the last vaccination, respectively.

#### 2.3.2. Immunogenicity

Evaluation of secondary immunogenicity endpoints included hemagglutination inhibition (HAI) geometric mean titers (GMTs) for each strain targeted by the study vaccine (i.e., homologous strains), HAI geometric mean fold rises (GMFRs) from before to 4 weeks after vaccination, and percentages of participants achieving HAI seroconversion for each strain 4 weeks after vaccination and with HAI titers ≥ 1:40 for each strain before and 4 weeks after vaccination. Methods for conducting the HAI assay have been reported previously [16].

Cell-mediated immune responses were assessed as an exploratory objective. To identify vaccine-elicited Th1 and/or Th2 type cytokine profiles of hemagglutinin-specific CD4+ and CD8+ T cells, T-cell responses were determined as GMFRs from before vaccination to 1 week and 4 weeks after vaccination by fluorescence-activated cell sorter (FACS)-based intracellular cytokine staining in a subset of participants as described previously [16]. In addition, interferon gamma (IFNγ) plus interleukin-2 (IL2) plus tumor necrosis factor alpha (TNFα) positive (IFNγ + IL2 + TNFα) and ≥ 1 Th1 cytokine responses from before to 1 week after vaccination were determined.

### 2.4. Statistical Analyses

The study sample size was not based on statistical hypothesis testing. For safety analyses, descriptive statistics are presented for binary variables.

Descriptive immunogenicity analyses were based on the evaluable immunogenicity populations, which included all participants who received study intervention as randomized, had valid and determinate assay results within 26 to 35 days after the last vaccination, and had no major protocol violations. The percentage of participants achieving HAI seroconversion at each time point after each vaccination, and with HAI titers ≥ 1:40 before each vaccination and 4 weeks after vaccination, are presented by vaccine group with associated 2-sided Clopper-Pearson 95% CIs for each strain and all strains. Seroconversion was defined as an HAI titer < 1:10 before vaccination and ≥ 1:40 after vaccination, or an HAI titer of ≥ 1:10 before vaccination with a 4-fold rise after vaccination. GMTs and GMFRs were calculated as the mean and the mean difference, respectively, of the assay results after logarithmic transformation and then exponentiation of the mean to express results on the original scale. Two-sided 95% CIs for GMTs and GMFRs were calculated using the mean (GMT) or the mean difference (GMFR) of the log-transformed assay results using the Student *t* distribution and then exponentiating the confidence limits.

## 3. Results

### 3.1. Phase 1 Substudy A

#### 3.1.1. Participants

A total of 436 participants were randomized and 434 were vaccinated at 19 US sites (Table S3). The majority were female, White, and non-Hispanic/non-Latino (Table S4).

#### 3.1.2. Safety

Pain at the injection site was the most frequently reported solicited local reaction and increased in a dose-dependent manner ranging from 25.0% to 64.3% in the mIRV groups and 31.3% to 60.0% in the bIRV groups; it was also 86.7% in the qIRV group and 51.7% in the overall QIV group. All solicited local reactions were mild or moderate in severity (Figure S1). Solicited systemic events also followed a general dose-related trend and were reported at similar frequencies in the mIRV and bIRV groups compared with the licensed QIV group (Figure S2). Fatigue and headache were the most common solicited systemic events across groups, and events were nearly all mild or moderate in severity. In the qIRV group, headaches and chills were reported at higher frequencies compared with the QIV group; all other solicited systemic events were reported at similar or lower frequencies in the qIRV versus the QIV group. Reactogenicity events were generally of short duration.

No participants discontinued due to unsolicited AEs; there were no confirmed cases of influenza, myocarditis, or pericarditis; and no new electrocardiogram (ECG) or laboratory findings were reported (Table S5).

#### 3.1.3. Immunogenicity

All IRVs investigated in substudy A (mIRV, bIRV, and qIRV) elicited HAI responses for their respective strain(s) (Figures S3–S5), with comparable results observed for qIRV and the licensed QIV control (Figure S3A). Peak HAI antibody responses were observed 1 to 4 weeks after vaccination in IRV groups and among QIV recipients. Postvaccination HAI GMTs for homologous (i.e., vaccine-encoded) strains were substantially increased against influenza A strains and more modestly increased against influenza B strains. HAI GMTs in the IRV groups were generally similar to those in the QIV group. The results for geometric mean fold rises (Figure S3A) and percentage of participants with seroconversion and HAI titers ≥ 1:40 overall reflected this same pattern (Figure S3B).

Collectively, the safety, tolerability, and immunogenicity data from substudy A supported continued phase 2 clinical development of IRV in substudy B.

### 3.2. Phase 2 Substudy B

#### 3.2.1. Participants

**Figure 1:**
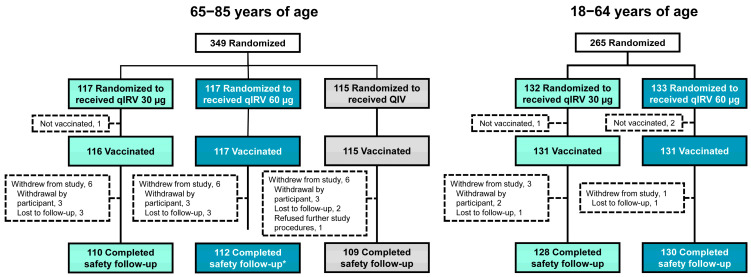
Participant disposition in substudy B. modRNA, nucleoside-modified messenger RNA; qIRV, quadrivalent influenza modRNA vaccine; QIV, quadrivalent influenza vaccine. * One participant of the 112 who completed the safety follow-up withdrew after vaccination but continued in the study for safety follow-up.

A total of 614 participants were randomized at 49 US sites, including 349 participants 65 through 85 years of age (116, 117, and 115 were vaccinated with qIRV 30 µg, qIRV 60 µg, and QIV, respectively) and 265 participants 18 through 64 years of age (131 each were vaccinated with qIRV 30 µg and qIRV 60 µg; Figure 1). Demographic characteristics were well balanced between groups (Table 1). The majority of participants were female, White, and Hispanic/Latino.

#### 3.2.2. Safety

**Figure 2:**
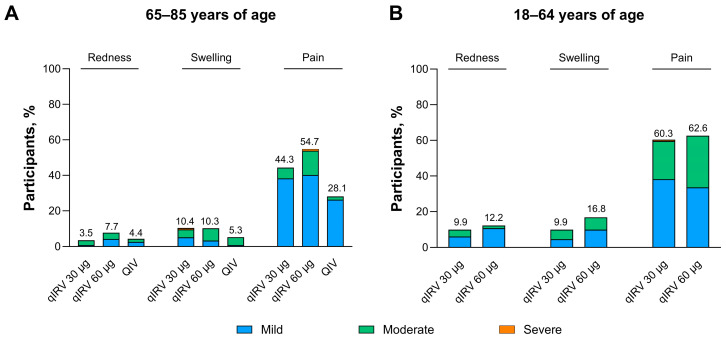
Local reactions occurring 7 days after vaccination among participants (**A**) 65 through 85 and (**B**) 18 through 64 years of age in substudy B. Data are for the safety population. The numbers above the bars are the percentage of participants in each group with each local reaction. The grading scale for local reactions is provided in Table S2. modRNA, nucleoside-modified messenger RNA; qIRV, quadrivalent influenza modRNA vaccine; QIV, quadrivalent influenza vaccine.

Solicited local reactions among participants 65 through 85 years of age and 18 through 64 years of age are displayed by type of reaction in Figure 2 and summarized further in Table S6. Pain at the injection site was the most common solicited local reaction, and most reactions were mild or moderate in severity. The onset of solicited local reactions was 1 to 2 days from the time of vaccination, and most resolved within 1 to 2 days. Among participants 65 through 85 years of age, incidences of solicited local reactions with qIRV 30 µg and 60 µg were similar or higher compared with licensed QIV. Among participants 18 through 64 years of age, a modest dose-dependent increase in swelling was observed between the qIRV 30 µg and qIRV 60 µg groups. Incidences of solicited local reactions with qIRV 30 µg and 60 µg were generally higher in participants 18 through 64 years of age compared with participants 65 through 85 years of age.

**Figure 3:**
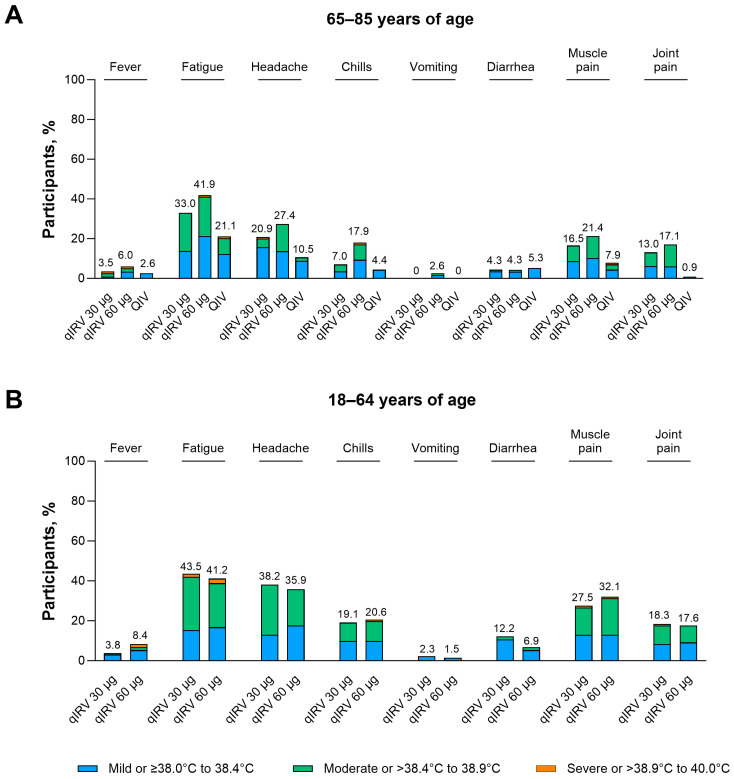
Systemic events occurring 7 days after vaccination among participants (**A**) 65 through 85 and (**B**) 18 through 64 years of age in substudy B. Data are for the safety population. The numbers above the bars are the percentage of participants in each group with each systemic event. The grading scale for systemic events is provided in Table S2. modRNA, nucleoside-modified messenger RNA; qIRV, quadrivalent influenza modRNA vaccine; QIV, quadrivalent influenza vaccine.

Solicited systemic events among participants 65 through 85 and 18 through 64 years of age are summarized in Figure 3 and Table S6. Fatigue and headache were the most common solicited systemic events, most solicited systemic events were mild or moderate in severity, and no grade 4 events or fever > 40 °C were reported. Across both age groups, the onset of solicited systemic events was within 1 to 2 days after vaccination, and most resolved within 1 to 2 days. In participants 65 through 85 years of age, incidences of solicited systemic events were similar or higher compared with licensed QIV. In participants 18 through 64 years of age, frequencies of solicited systemic events were similar between the qIRV 30 µg and 60 µg groups, apart from a dose-dependent increase in frequencies of fever and muscle pain. Frequencies of solicited systemic events associated with qIRV 30 µg were generally higher in participants 18 through 64 years of age when compared with participants 65 through 85 years of age, whereas those associated with qIRV 60 µg were similar between age groups.

In participants 65 through 85 years of age, unsolicited AEs were reported by 6.9%, 8.5%, and 7.0% of participants receiving qIRV 30 µg, qIRV 60 µg, and QIV, respectively (Table S6). In participants 18 through 64 years of age, unsolicited AEs were reported by 1.5% and 3.1% of participants receiving qIRV 30 µg and qIRV 60 µg, respectively. The majority of AEs in both age groups were mild or moderate in severity. No severe events in either age group were considered related to the study vaccine. In participants 65 through 85 years of age, one SAE was reported (osteoarthritis worsening in a qIRV 30 µg recipient). In participants 18 through 64 years of age, two participants experienced SAEs (pneumonia, sepsis, and viral infection in one qIRV 60 µg recipient, and hip fracture in a second qIRV 60 µg recipient). No SAEs in either age group were considered related to the study vaccine. Across both age groups, neither AEs leading to discontinuation nor deaths were reported.

In participants 65 through 85 years of age, vaccine-related AEs were reported by 1.7% of participants in each of the three vaccine groups (Table S6). Two of the events represent local vaccine-associated reactions (injection site pruritus and hemorrhage subcutaneous, both in the qIRV 30 µg group) and two additional events relating to vestibular findings (tinnitus and dizziness) were reported in the qIRV 60 µg group and QIV groups, respectively. Of note, one 65-year-old participant with a history of viral infection and illicit drug use who received qIRV 60 µg had abnormal ECG findings and mildly elevated troponin levels 7 days after vaccination; these were considered by the investigator to be consistent with myocarditis or pericarditis and related to study vaccination. Given the participant’s recent medical history and illicit drug use, the sponsor’s assessment was that the event was unlikely related to study vaccine. In participants 18 through 64 years of age, no related unsolicited AEs were reported by those receiving qIRV 30 µg or qIRV 60 µg.

#### 3.2.3. Immunogenicity

**Figure 4:**
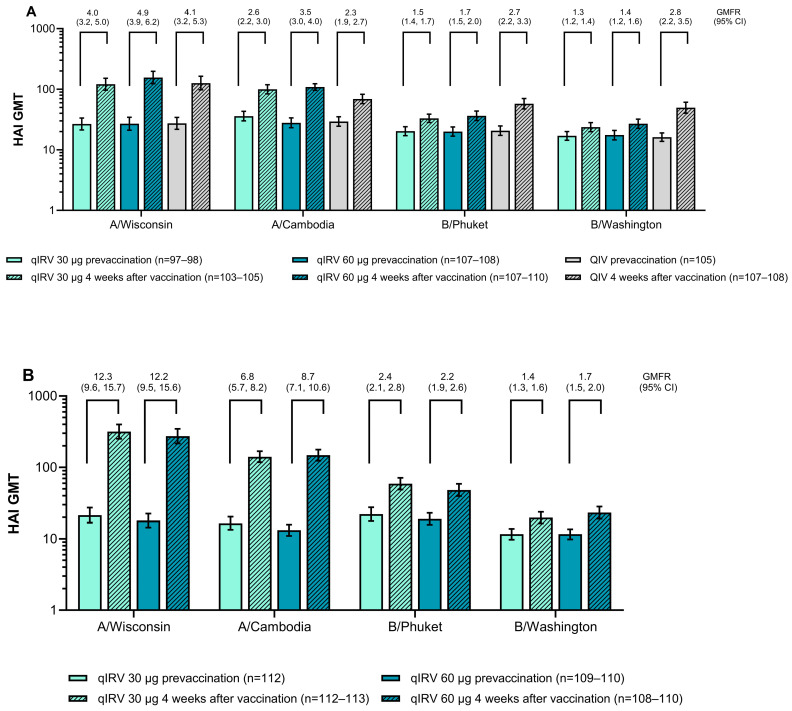
HAI GMTs and GMFRs 4 weeks after vaccination among participants (**A**) 65 through 85 and (**B**) 18 through 64 years of age in substudy B. Results are for the evaluable immunogenicity population. GMTs and GMFRs were calculated by exponentiating the logarithmic mean or mean of the fold rises, respectively, with corresponding and two-sided 95% CIs based on the Student *t* distribution. Assay results below the LLOQ were set to 0.5 × LLOQ. GMFR, geometric mean fold rise, GMT, geometric mean titer; HAI, hemagglutination inhibition; LLOQ, lower limit of quantitation; modRNA, nucleoside-modified messenger RNA; qIRV, quadrivalent influenza modRNA vaccine; QIV, quadrivalent influenza vaccine.

Among all participants 18 through 85 years of age, there was an increase in HAI GMTs after vaccination versus before vaccination (Figure 4). Among participants 65 through 85 years of age, HAI GMTs against influenza A strains were higher in the qIRV 60 µg group compared with QIV, but lower for influenza B strains at 4 weeks after vaccination (Figure 4A). In addition, HAI GMTs for the qIRV 30 µg group were similar (A/Wisconsin (i.e., A/H1N1)) or higher (A/Cambodia (i.e., A/H3N2)) compared with the QIV group, but lower for influenza B strains. The qIRV 60 µg group titers trended similar or higher compared with the qIRV 30 µg group for all four influenza strains. Among participants 18 through 64 years of age, postvaccination HAI GMTs were generally similar in the qIRV 30 μg and 60 μg groups, and increases after vaccination were most pronounced against influenza A strains (Figure 4B). HAI GMTs were generally higher in the younger versus older adults who received qIRV 30 μg or qIRV 60 μg, except for HAI GMTs for B/Washington (i.e., B/Victoria), which were similar in both age groups.

**Figure 5:**
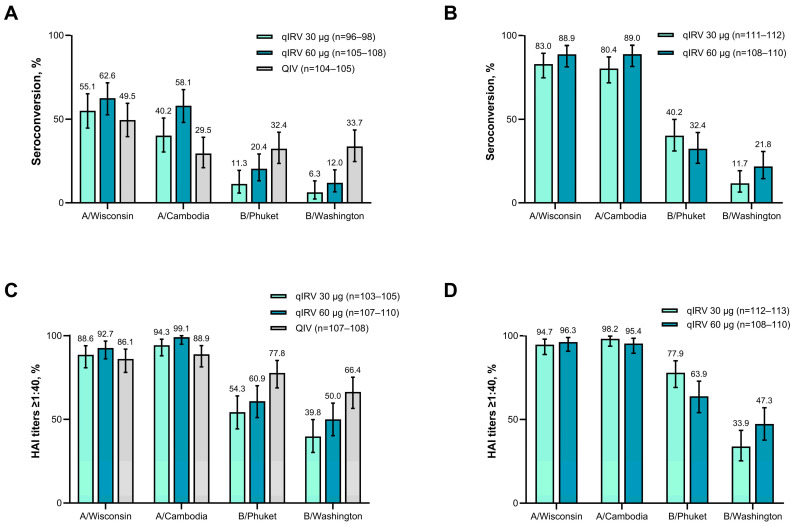
Percentage of participants with seroconversion 4 weeks after vaccination among participants (**A**) 65 through 85 and (**B**) 18 through 64 years of age, and the percentage of participants achieving postvaccination HAI titers ≥ 1:40 at 4 weeks after vaccination among participants (**C**) 65 through 85 and (**D**) 18 through 64 years of age in substudy B. Results are for the evaluable immunogenicity population. Seroconversion was defined as an HAI titer < 1:10 before the first vaccination and ≥ 1:40 at the timepoint of interest, or an HAI titer of ≥ 1:10 before the first vaccination with a four-fold rise at the timepoint of interest. Exact two-sided CIs are based on the Clopper-Pearson method. HAI, hemagglutination inhibition; modRNA, nucleoside-modified messenger RNA; qIRV, quadrivalent influenza modRNA vaccine; QIV, quadrivalent influenza vaccine.

At 4 weeks after vaccination, seroconversion against both influenza A strains occurred in a greater percentage of participants 65 through 85 years of age in both qIRV groups (30 µg and 60 µg) compared with the QIV group, with the highest percentages in the qIRV 60 µg group (Figure 5A). The percentage of participants 65 through 85 years of age with seroconversion against both influenza B strains was higher in the QIV group compared with both qIRV groups, and higher in the qIRV 60 µg group than in the qIRV 30 µg group for all four influenza strains. The percentage of participants 65 through 85 years of age who achieved HAI titers ≥ 1:40 at 4 weeks after vaccination showed a similar pattern (Figure 5C).

In participants 18 through 64 years of age, seroconversion rates and percentages of participants who achieved HAI titers ≥ 1:40 after vaccination were not consistent across dose levels. Seroconversion rates against each influenza strain at 4 weeks after vaccination were generally higher in the qIRV 60 μg group compared with the qIRV 30 μg group except for B/Phuket (i.e., B/Yamagata), which was lower in the qIRV 60 μg group (Figure 5B). The percentage of participants with HAI titers ≥ 1:40 at 4 weeks after vaccination was higher in the qIRV 60 μg group compared with the qIRV 30 μg group (except for A/Cambodia and B/Phuket in participants 18 through 64 years of age; Figure 5D).

**Figure 6:**
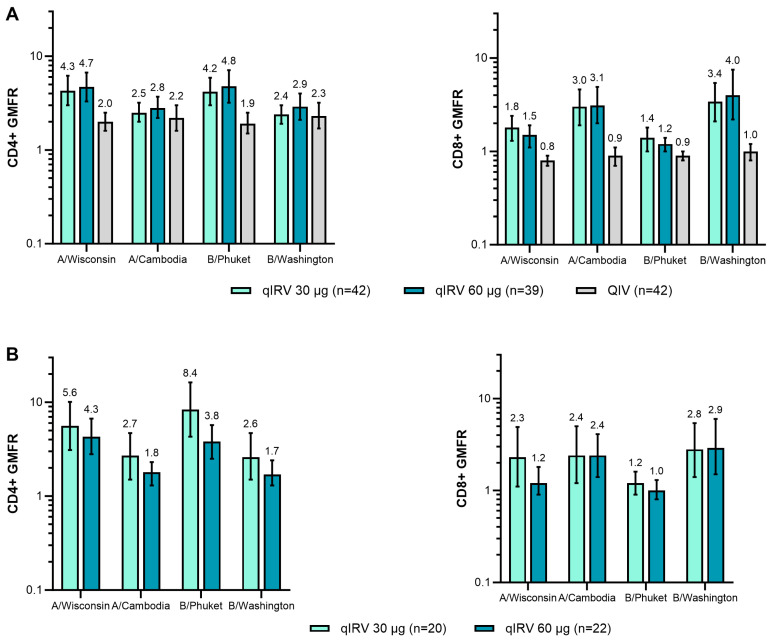
IFNγ+ (% of CD4) and IFNγ+ (% of CD8) T-cell responses of PBMCs at 1 week after vaccination in participants (**A**) 65 through 85 and (**B**) 18 through 64 years of age in substudy B. Results are for the evaluable immunogenicity population. ICS assay LLOQ values were: IFNγ+ (% of CD4) = 0.00260 and IFNγ+ (% of CD8) = 0.01636. GMFRs and corresponding two-sided 95% CIs were calculated by exponentiating the mean logarithm of the fold rises and the corresponding CIs (based on the Student *t* distribution). Assay results < LLOQ or < LOD were set to 0.5 × LLOQ. GMFR, geometric mean fold rise; ICS, intracellular cytokine staining; IFNγ, interferon gamma; LLOQ, lower limit of quantitation; LOD, limit of detection; modRNA, nucleoside-modified messenger RNA; PBMC, peripheral blood mononuclear cell; qIRV, quadrivalent influenza modRNA vaccine; QIV, quadrivalent influenza vaccine.

In participants 65 through 85 years of age, cell-mediated immune responses to the IRVs at 1 week after vaccination showed overall higher CD4+ and CD8+ T-cell responses against all four strains compared with QIV, and T-cell responses were similar or higher after qIRV 60 μg compared with after qIRV 30 μg (Figure 6; Figure S6). CD4+ and CD8+ T-cell responses generally peaked at 1 week after vaccination, and plateaued or contracted at 4 weeks after vaccination (Figure S7). The T-cell GMFRs were generally greater or similar in the qIRV groups compared with the licensed QIV control group for all four influenza strains. In participants 18 through 64 years of age, the CD4+ and CD8+ T-cell responses were similar in both qIRV dose groups (Figure 6).

## 4. Discussion

Because of the ongoing variability in circulating influenza viruses, a highly compressed schedule for vaccine production, release, and administration is necessary [7,8]. The modRNA vaccine platform offers a means of economic, rapid, large-scale vaccine production [11,19], while presenting opportunities to reduce the probability of antigenic differences between vaccine antigens and circulating influenza strains [12]. Therefore, the modRNA platform for influenza vaccines was assessed in this phase 1/2 study in healthy adults 18 through 85 years of age. In phase 1 of this study, multiple vaccines of different valencies were studied in a small number of participants to identify groups for further investigation in phase 2, which aimed to identify the vaccine for assessment in later-stage clinical trials.

Findings from phase 1 of the study supported assessment of qIRV 30 µg and 60 µg in phase 2 of the study (substudy B), in which similar or higher antibody responses and T-cell responses to all four homologous strains were observed in participants 65 through 85 years of age elicited by a single dose of qIRV 60 µg compared with qIRV 30 µg. Among this age group, despite lower antibody responses of qIRV compared with the licensed QIV control for the B strains (as measured by HAI GMTs, GMFRs, and seroconversion), higher IFNγ-producing CD4+ and CD8+ T-cell responses to all four influenza strains were elicited following qIRV compared to licensed QIV. In both age groups, the CD4+ and CD8+ GMFR increases observed after vaccination did not show meaningful differences between the two dose levels of qIRV. Percentages of participants with seroconversion trended similarly between qIRV 60 µg and 30 µg for all four influenza strains. Single doses of qIRV 60 µg and 30 µg were well tolerated in this age group, and both dose levels showed a consistent reactogenicity profile to that of COVID-19 modRNA vaccines [15]. The safety profile of both dose levels also reflected events that may be expected in the general population for this group of older adults and suggested no new safety concerns with modRNA-containing influenza vaccines. Considering the higher risk of severe complications of influenza as well as the improved immune response with the higher dose, a single dose of qIRV 60 µg was selected for further evaluation in participants ≥ 65 years of age in the pivotal phase 3 efficacy study.

Antibody and T-cell responses in participants 18 through 64 years of age were elicited at both dose levels (qIRV 30 µg and qIRV 60 µg), while a modest dose-dependent increase in reactogenicity was observed between the two dose levels. Reactogenicity incidences associated with qIRV 30 µg were also generally higher in participants 18 through 64 years of age when compared with those 65 through 85 years of age, which is expected, as older adults are known to report less reactogenicity events than younger adults [20]. At the qIRV 60-µg dose level, incidences of local reactions were higher in the younger age group compared with the older age group, whereas incidences of systemic events were similar. In addition, the safety profile of qIRV at both dose levels reflected expected events in 18- through 64-year-olds and suggested no new safety concerns. Based on the totality of data in this study, a single dose of qIRV 30 µg was selected for further evaluation against licensed QIV in participants 18 through 64 years of age in the phase 3 efficacy study.

Influenza vaccination is intended to induce antibodies against the viral surface protein hemagglutinin, thereby neutralizing the infectivity of the influenza virus by preventing binding of the virus to the host cell [21,22]. By measuring strain-specific immunogenicity, the HAI assay assesses the ability of serum antibodies to inhibit agglutination between erythrocytes and viral hemagglutinin, with HAI titers ≥ 1:40 used as the primary immunologic correlate of protection against influenza infection and to support licensure [22,23]. Limitations of the HAI assay include its reported underperformance in detecting responses to influenza B strains as described previously, with variability in HAI titers for influenza B strains associated with age, titer levels before infection, and specific influenza B lineage [24,25,26,27]. This is consistent with observations from our study, in which HAI responses to qIRV against influenza A strains were generally higher than responses against influenza B strains for both age groups.

The HAI responses to qIRV in our study were also generally higher in younger versus older adults, except for B/Washington, which were similar in both age groups. Notably, substantially higher protective levels of HAI titers are required in older adults compared with younger healthy adults [22]. This may be partially attributed to immunosenescence causing an age-associated decrease in the functioning of B and T cells, necessitating a more balanced humoral and cellular response to provide protection in the elderly compared to younger adults who may only require humoral immune responses for protection against influenza.

To compensate for weakened humoral immunity in older adults, strong vaccine-induced T-cell responses may be needed to confer protection against influenza, particularly cell-mediated immunity derived from CD8+ T cells, which are critical for decreasing viral load by killing virus-infected cells [22]. Compared with humoral immunity, cell-mediated immunity against influenza, which is more cross-reactive, has the potential to protect against multiple strains and to provide a broad response to vaccination, including among the elderly. As shown in earlier COVID-19 vaccine trials, modRNA vaccines have the potential to provide protection through both antibody and T-cell responses [13].

In our study, qIRV-elicited T-cell responses were observed across age and dose level groups and reflected both induction of CD8+ T cells and a Th1-type CD4+ T-cell response. This Th1-type cytokine profile, characterized by vaccine-elicited IFNγ production in the absence of IL-4 induction, is associated with effective antiviral immunity. T-cell responses were generally similar against both influenza A strains and B strains, in contrast to the difference in responses between strains observed for HAI titers. It is expected that both humoral responses (i.e., HAI titers) and cell-mediated responses can contribute to the ability of qIRV to broadly protect against influenza disease, which may be particularly relevant in older populations [22].

This trial has limitations. The small sample sizes limit interpretation of findings across groups, including a limited subset of participants evaluated for cell-mediated immune responses. The study was also conducted in the United States in a predominantly White and healthy population, potentially limiting generalizability to other populations. The long-term persistence of antibody responses was also not elucidated. In addition, a QIV comparator was not included among the group of participants 18 through 64 years of age assessed in phase 2. However, the immunogenicity, relative vaccine efficacy, and safety and tolerability of qIRV at the 30-µg and 60-µg dose levels have been assessed in a pivotal phase 3 efficacy trial in participants 18 years and older compared with QIV, and results will be published separately.

In conclusion, the totality of the safety, tolerability, and immunogenicity data analyzed in this phase 1/2 study demonstrated that single dose modRNA qIRV 30 µg or 60 µg was well tolerated, raised no new safety concerns, and elicited immune responses against all four vaccine-encoded influenza strains in participants 18 through 85 years of age. These data supported phase 3 clinical investigations of qIRV 60 µg in older adults and qIRV 30 µg in younger adults.

## Figures and Tables

**Table 1:** Participant demographics in substudy B.

	65–85 Years of Age			18–64 Years of Age	
	qIRV 30 μg N = 116	qIRV 60 μg N = 117	Licensed QIV N = 115	qIRV 30 μg N = 131	qIRV 60 μg N = 131
Sex, *n* (%)					
Male	55 (47.4)	57 (48.7)	48 (41.7)	54 (41.2)	57 (43.5)
Female	61 (52.6)	60 (51.3)	67 (58.3)	77 (58.8)	74 (56.5)
Age at vaccination, mean (SD), years	71.4 (5.03)	71.9 (5.05)	71.6 (4.59)	42.8 (13.52)	44.7 (12.91)
Race, *n* (%)					
White	92 (79.3)	101 (86.3)	96 (83.5)	98 (74.8)	107 (81.7)
Black	21 (18.1)	12 (10.3)	14 (12.2)	20 (15.3)	20 (15.3)
Asian	1 (0.9)	2 (1.7)	1 (0.9)	6 (4.6)	1 (0.8)
Native Hawaiian, other Pacific Islander, American Indian, or Alaska native	2 (1.7)	0	1 (0.9)	1 (0.8)	1 (0.8)
Multiracial	0	1 (0.9)	3 (2.6)	4 (3.1)	0
Not reported	0	1 (0.9)	0	2 (1.5)	2 (1.5)
Ethnicity, *n* (%)					
Hispanic/Latino	50 (43.1)	60 (51.3)	55 (47.8)	74 (56.5)	77 (58.8)
Non-Hispanic/non-Latino	65 (56.0)	56 (47.9)	60 (52.2)	57 (43.5)	54 (41.2)
Not reported	1 (0.9)	1 (0.9)	0	0	0

modRNA, nucleoside-modified messenger RNA; qIRV, quadrivalent influenza modRNA vaccine; QIV, quadrivalent influenza vaccine. Data are for the safety population.

## Data Availability

Upon request, and subject to review, Pfizer will provide the data that support the findings of this study. Subject to certain criteria, conditions, and exceptions, Pfizer may also provide access to the related individual de-identified participant data. See https://www.pfizer.com/science/clinical-trials/trial-data-and-results for more information (accessed on 30 March 2025).

## Supplementary Materials

The following supporting information can be downloaded at: https://www.mdpi.com/article/10.3390/vaccines13040383/s1, Supplementary Text S1: Design of substudy A; Eligibility criteria of substudy A; Eligibility criteria of substudy B; List of principal investigators; Figure S1: Local reactions occurring within 7 days after vaccination 1 in substudy A; Figure S2: Systemic events occurring within 7 days after vaccination 1 in substudy A; Figure S3: HAI GMTs (A) and percentage of participants with seroconversion and HAI titers ≥ 1:40 (B) for qIRV 30 µg and QIV in substudy A; Figure S4: HAI GMTs and GMFRs for (A) mIRV and (B) bIRV before and 4 weeks after vaccination in substudy A; Figure S5: Percentage of participants with seroconversion for (A) mIRV and (B) bIRV and with HAI titers ≥ 1:40 for (C) mIRV and (D) bIRV) at 4 weeks after vaccination in substudy A; Figure S6: IFNγ + IL2+ TNFα+ (% of CD4) and ≥ 1 TH1 cytokine (% of CD4) T-cell responses (geometric mean fold rises) of PBMCs 1 week after vaccination in participants (A) 65 through 85 and (B) 18 through 64 years of age in substudy B; Figure S7: IFNγ+ (% of CD4) and IFNγ+ (% of CD8) T-cell responses of PBMCs at 4 weeks after vaccination in participants (A) 65 through 85 and (B) 18 through 64 years of age in substudy B; Table S1: Strain selections; Table S2: Grading scale for local reactions and systemic events; Table S3: Participant disposition in substudy A; Table S4: Participant demographics in substudy A; Table S5: Summary of adverse events in substudy A; Table S6: Summary of solicited local reactions and systemic events and unsolicited adverse events in substudy B.
