# Supplementary material for: A Phase 1/2 Randomized Study to Evaluate the Safety, Tolerability, and Immunogenicity of Nucleoside-Modified Messenger RNA Influenza Vaccines in Healthy Adults
Source: Vaccines (Basel). 2025 Apr 3;13(4):383. doi: 10.3390/vaccines13040383 (PMC12031420; doi:10.3390/vaccines13040383)

Figure S1. Local reactions occurring within 7 days after vaccination 1 in substudy A

The number of participants per group was 14–16 (mIRV-A), 14–17 (mIRV-B), 14–16 (bIRV), 15 (qIRV), and 60 (QIV). bIRV-A+B, bivalent influenza modRNA vaccine containing 1 A and 1 B strain antigen; modRNA, nucleoside-modified messenger RNA; mIRV-A, monovalent influenza modRNA vaccine containing 1 A strain antigen; mIRV-B, monovalent influenza modRNA vaccine containing 1 B strain antigen; qIRV, quadrivalent influenza modRNA vaccine; QIV, quadrivalent influenza vaccine.

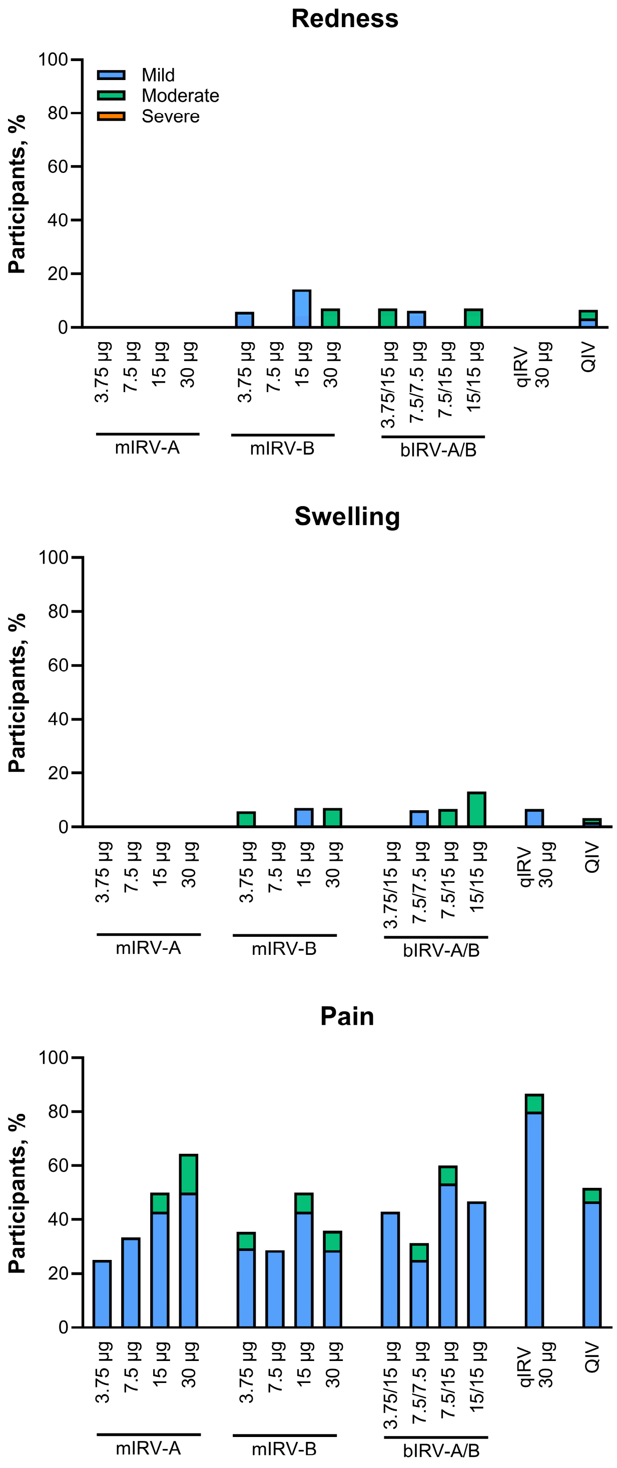

Supplement: Supplementary file 1 [file vaccines-13-00383-s001.zip › Branche_Figure S1.pdf]
