# Supplementary material for: A Phase 1/2 Randomized Study to Evaluate the Safety, Tolerability, and Immunogenicity of Nucleoside-Modified Messenger RNA Influenza Vaccines in Healthy Adults
Source: Vaccines (Basel). 2025 Apr 3;13(4):383. doi: 10.3390/vaccines13040383 (PMC12031420; doi:10.3390/vaccines13040383)

Figure S3. HAI GMTs (A) and percentage of participants with seroconversion and HAI titers ≥1:40 (B) for qIRV 30 μg and QIV in substudy A

Results are for the evaluable immunogenicity population. GMTs were calculated by exponentiating the logarithmic mean with corresponding and 2-sided 95% CIs based on the Student *t* distribution. Assay results below the LLOQ were set to 0.5 × LLOQ. GMFRs are from before to 4 weeks after vaccination. Seroconversion was defined as an HAI titer <1:10 before the first vaccination and ≥1:40 at the timepoint of interest, or an HAI titer of ≥1:10 before the first vaccination with a 4-fold rise at the timepoint of interest. N values are 14 and 15–40 for the qIRV 30 μg and QIV group, respectively. GMT, geometric mean titer; GMFR, geometric mean fold rise; HAI, hemagglutination inhibition; LLOQ, lower limit of quantitation; modRNA, nucleoside-modified messenger RNA; qIRV, quadrivalent influenza modRNA vaccine; QIV, quadrivalent influenza vaccine.

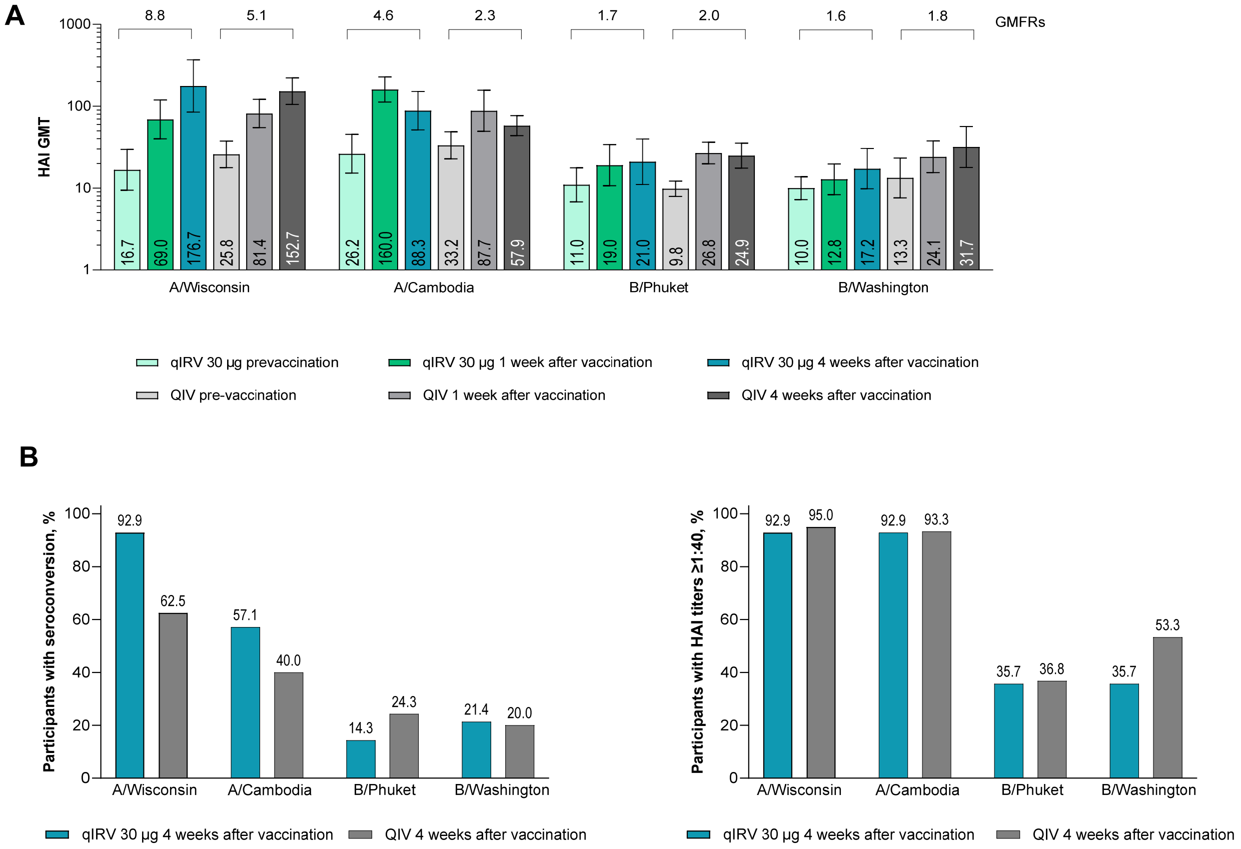

Supplement: Supplementary file 1 [file vaccines-13-00383-s001.zip › Branche_Figure S3.pdf]
