# Supplementary material for: A Phase 1/2 Randomized Study to Evaluate the Safety, Tolerability, and Immunogenicity of Nucleoside-Modified Messenger RNA Influenza Vaccines in Healthy Adults
Source: Vaccines (Basel). 2025 Apr 3;13(4):383. doi: 10.3390/vaccines13040383 (PMC12031420; doi:10.3390/vaccines13040383)

Figure S4. HAI GMTs and GMFRs for (A) mIRV and (B) bIRV before and 4 weeks after vaccination in substudy A

Results are for the evaluable immunogenicity population after the first vaccination. GMTs and GMFRs were calculated by exponentiating the logarithmic mean or mean fold rise, respectively, with corresponding and 2-sided 95% CIs based on the Student *t* distribution. Assay results below the LLOQ were set to 0.5 × LLOQ. GMFRs are from before to 4 weeks after vaccination. bIRV, bivalent influenza modRNA vaccine; GMFR, geometric mean fold rise; GMT, geometric mean titer; HAI, hemagglutination inhibition; LLOQ, lower limit of quantitation; modRNA, nucleoside-modified messenger RNA; mIRV, monovalent influenza modRNA vaccine.

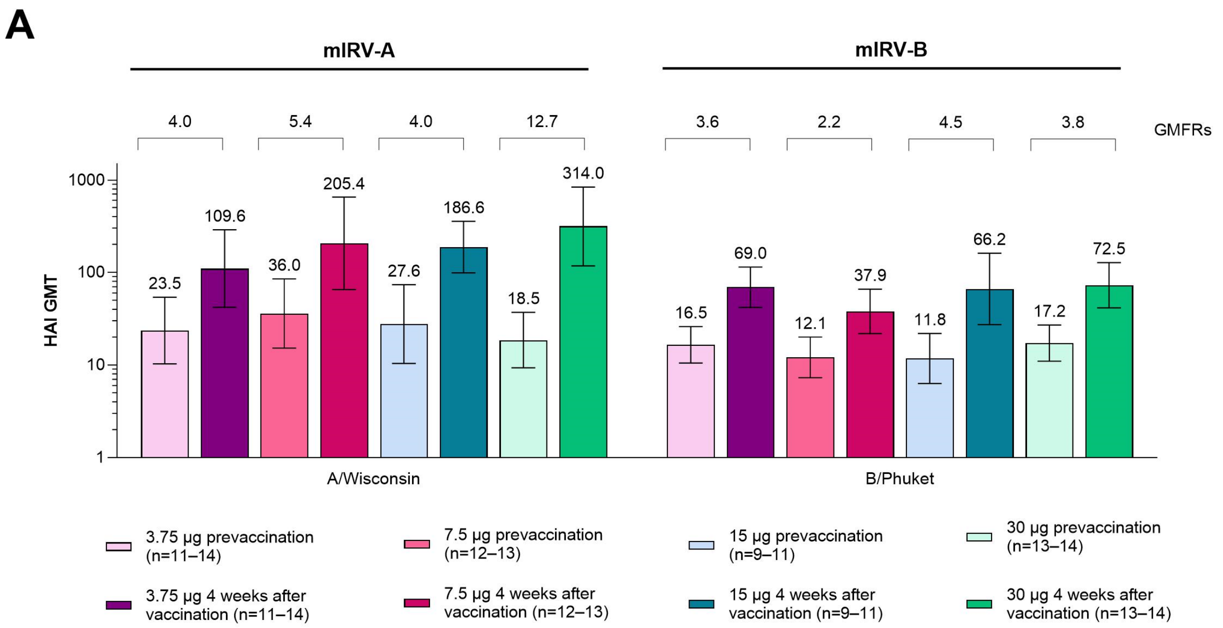

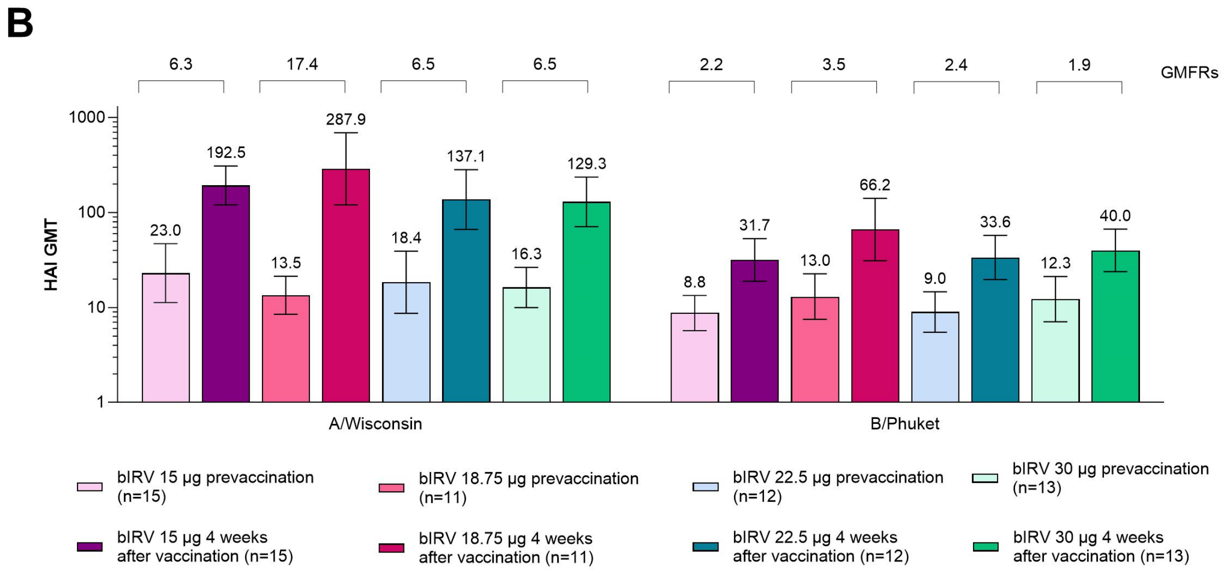

Supplement: Supplementary file 1 [file vaccines-13-00383-s001.zip › Branche_Figure S4.pdf]
