# Supplementary material for: A Phase 1/2 Randomized Study to Evaluate the Safety, Tolerability, and Immunogenicity of Nucleoside-Modified Messenger RNA Influenza Vaccines in Healthy Adults
Source: Vaccines (Basel). 2025 Apr 3;13(4):383. doi: 10.3390/vaccines13040383 (PMC12031420; doi:10.3390/vaccines13040383)

Figure S5. Percentage of participants with seroconversion for (A) mIRV and (B) bIRV and with HAI titers ≥1:40 for (C) mIRV and (D) bIRV) at 4 weeks after vaccination in substudy A

Results are for the evaluable immunogenicity population after the first vaccination. Seroconversion was defined as an HAI titer <1:10 before the first vaccination and ≥1:40 at the timepoint of interest, or an HAI titer of ≥1:10 before the first vaccination with a 4-fold rise at the timepoint of interest. bIRV, bivalent influenza modRNA vaccine; HAI, hemagglutination inhibition; modRNA, nucleoside-modified messenger RNA; mIRV, monovalent influenza modRNA vaccine.

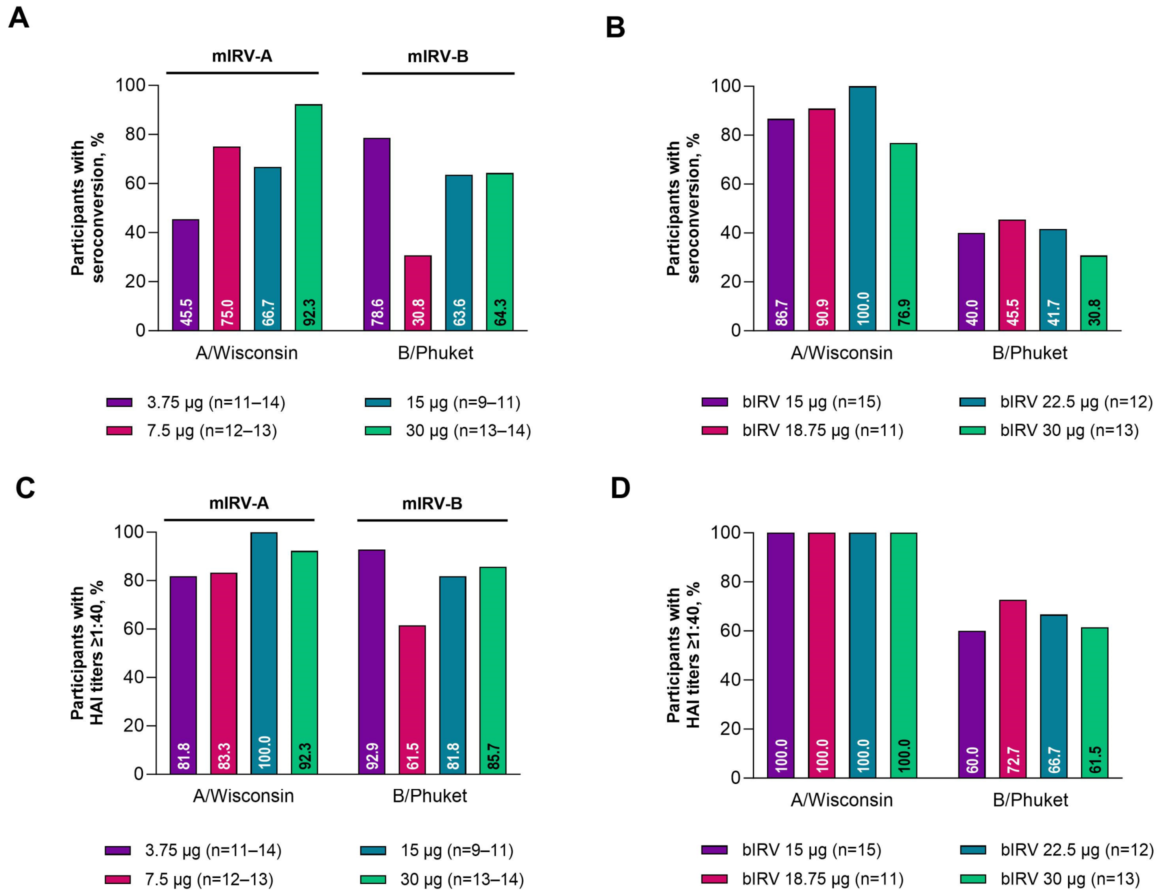

Supplement: Supplementary file 1 [file vaccines-13-00383-s001.zip › Branche_Figure S5.pdf]
