# Supplementary material for: A Phase 1/2 Randomized Study to Evaluate the Safety, Tolerability, and Immunogenicity of Nucleoside-Modified Messenger RNA Influenza Vaccines in Healthy Adults
Source: Vaccines (Basel). 2025 Apr 3;13(4):383. doi: 10.3390/vaccines13040383 (PMC12031420; doi:10.3390/vaccines13040383)

Figure S6. IFNγ + IL2+ TNFα+ (% of CD4) and ≥1 TH1 cytokine (% of CD4) T-cell responses (geometric mean fold rises) of PBMCs 1 week after vaccination in participants (A) 65 through 85 and (B) 18 through 64 years of age in substudy B

Results are for the evaluable immunogenicity population. ICS assay LLOQ values were: IFNγ +IL2+ TNFα+ (% of CD4) = 0.01414, ≥1 TH1 cytokine (% of CD4) = 0.01414. GMFRs and corresponding 2-sided 95% CIs based on the Student *t* distribution were calculated by exponentiating the mean logarithm of the fold rises and the corresponding CIs. Assay results <LLOQ or <LOD were set to 0.5 × LLOQ. ICS, intracellular cytokine staining; IFNγ, interferon gamma; IL2, interleukin-2; LLOQ, lower limit of quantitation; LOD, limit of detection; modRNA, nucleoside-modified messenger RNA; PBMC, peripheral blood mononuclear cell; qIRV, quadrivalent influenza modRNA vaccine; QIV, quadrivalent influenza vaccine, TH1, T helper 1.

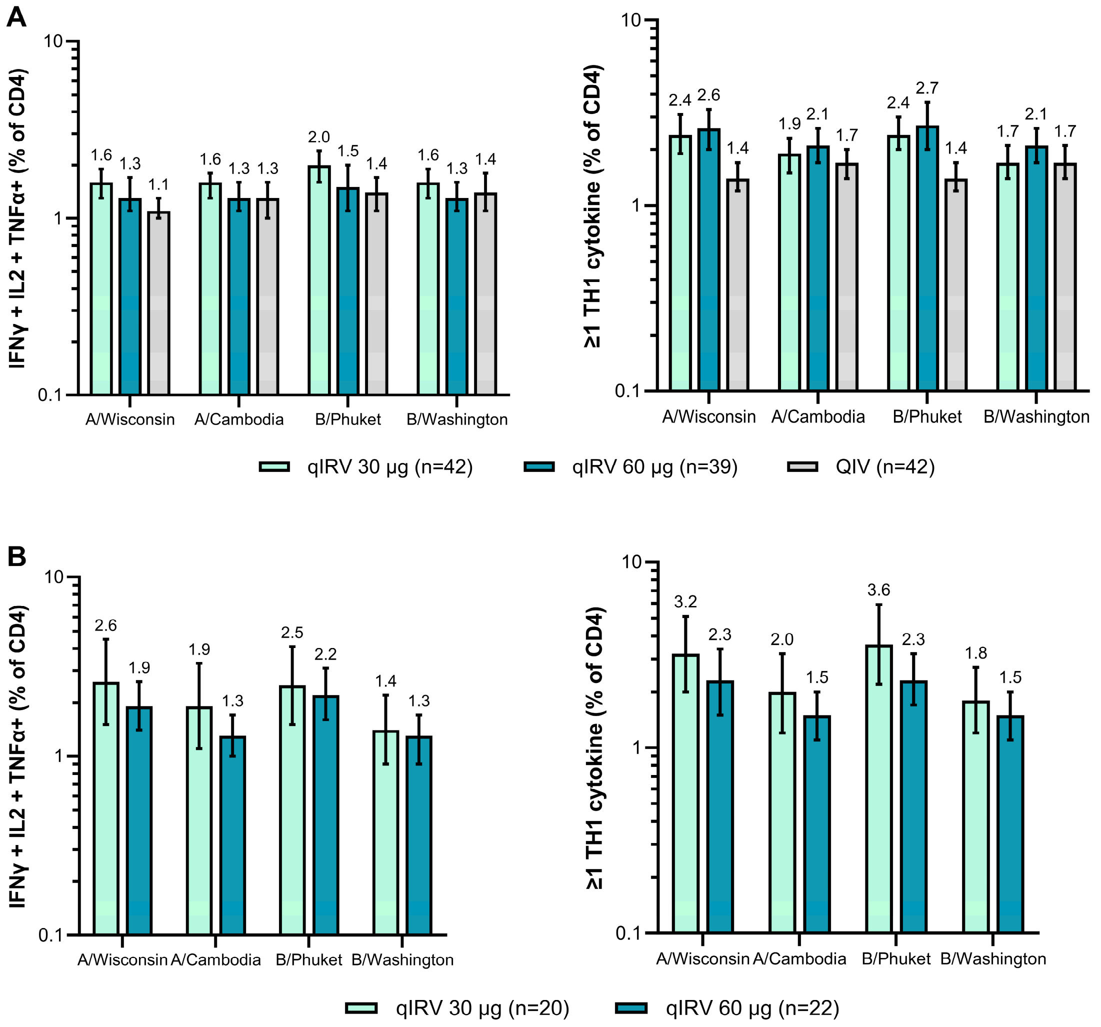

Supplement: Supplementary file 1 [file vaccines-13-00383-s001.zip › Branche_Figure S6.pdf]
