# Supplementary material for: A Phase 1/2 Randomized Study to Evaluate the Safety, Tolerability, and Immunogenicity of Nucleoside-Modified Messenger RNA Influenza Vaccines in Healthy Adults
Source: Vaccines (Basel). 2025 Apr 3;13(4):383. doi: 10.3390/vaccines13040383 (PMC12031420; doi:10.3390/vaccines13040383)
Supplement: Supplementary file 1 [file vaccines-13-00383-s001.zip › Branche_Supplementary Text S1.pdf]

SUPPLEMENTARY TEXT

Design of substudy A

In substudy A, participants were randomized to receive one of the following:

- Monovalent influenza modRNA vaccine (mIRV) encoding strain A (mIRV-A; 3.75, 7.5, 15, or 30 μg), mIRV encoding strain B (mIRV-B; 3.75, 7.5, 15, or 30 μg), or licensed quadrivalent influenza vaccine (QIV).
- Bivalent IRV (bIRV; encoding combinations of A [bIRV-A] strain [3.75, 7.5, or 15 μg] and B [bIRV-B] strain [7.5 or 15 μg]) or QIV as a control.
- Quadrivalent IRV (qIRV; encoding 2 A strains and 2 B strains; 7.5 μg per strain) or QIV.

Initially, 15 participants per group (ie, formulation, dose level, or control) were enrolled; this was expanded after a review of safety. Eight weeks after vaccination, QIV was administered to participants who did not previously receive it. Participants who previously received QIV received either mIRV-A 30 μg or mIRV-B 30 μg.

Eligibility criteria of substudy A

Inclusion criteria were:

- Male or female participants 65 through 85 years of age.
- Willing and able to comply with all scheduled visits, vaccination plan, laboratory tests, lifestyle restrictions, and other study procedures.
- Healthy as determined by medical history, physical examination (if required), and clinical judgment of the investigator (healthy participants with preexisting stable disease, defined as disease not requiring significant change in therapy or hospitalization for worsening disease during the 6 weeks before enrollment could be included).
- Male participant able to father children and willing to use an acceptable method of contraception for ≥28 days after the last dose of study intervention; or female participant not of childbearing potential; or male participant not able to father children.
- Capable of giving signed informed consent.

Exclusion criteria were:

- Medical conditions
  - Other medical or psychiatric condition including recent (within the past year) or active suicidal ideation/behavior or laboratory abnormality that may increase the risk of study participation or make the participant inappropriate for the study.
  - History of severe adverse reaction associated with a vaccine and/or severe allergic reaction (eg, anaphylaxis) to any component of the study intervention(s).
  - Immunocompromised individuals with known or suspected immunodeficiency, as determined by history and/or laboratory/physical examination.

    - Bleeding diathesis or condition associated with prolonged bleeding that would contraindicate intramuscular injection.
    - Pregnant or breastfeeding.
    - Allergy to egg proteins (egg or egg products) or chicken proteins.
- Prior/concomitant therapy
    - Having significant exposure (someone who was within 6 feet of an infected person for a cumulative total of 15 minutes or more over a 24-hour period) to someone with laboratory-confirmed SARS-CoV-2 infection, COVID-19, or influenza in the past 14 days known before the first study visit.
    - Having SARS-CoV-2 RT-PCR or antigen test in the past 10 days before the first study visit that has not been confirmed as negative.
    - Receiving treatment with radiotherapy or immunosuppressive therapy, including cytotoxic agents or systemic corticosteroids (if systemic corticosteroids are administered for ≥14 days at a dose of ≥20 mg/day of prednisone or equivalent), eg, for cancer or an autoimmune disease, or planned receipt throughout the study (inhaled/nebulized, intra-articular, intrabursal, or topical [skin or eyes] corticosteroids were permitted).
    - Receipt of blood/plasma products, immunoglobulin, or monoclonal antibodies, from 60 days before study intervention administration, or planned receipt throughout the study.
    - Vaccination with any influenza vaccine within 6 months (175 days) before study intervention administration.
    - Any participant who received or planned to receive a modRNA-platform SARS-CoV-2 vaccine within 60 days of the first study visit.
- Other exclusions
    - Participation in other studies involving study intervention within 28 days before study entry and/or during study participation.
    - Any screening hematology and/or blood chemistry laboratory value meeting the definition of a ≥grade 1 abnormality, or an abnormal C-reactive protein or troponin I value (except for bilirubin, participants with any stable Grade 1 abnormalities according to the toxicity grading scale were considered eligible at the discretion of the investigator, with a "stable" Grade 1 laboratory abnormality defined as a report of Grade 1 on an initial blood sample that remains ≤Grade 1 upon repeat testing on a second sample from the same participant.)
    - Screening 12-lead electrocardiogram (ECG) that was consistent with probable or possible myocarditis or pericarditis, or demonstrated clinically relevant abnormalities that may have affected participant safety or interpretation of study results (eg, QTcF interval >450 msec, complete left bundle branch block, signs of an acute or indeterminate-age myocardial infarction, ST-T interval changes suggestive of myocardial ischemia, second- or third-degree AV block, or serious bradyarrhythmias or tachyarrhythmias).
    - Investigator site staff or sponsor employees directly involved in the conduct of the study, site staff otherwise supervised by the investigator, and their respective family members.
    - Participation in strenuous or endurance exercise throughout the study.

- History of heart disease.

Eligibility criteria of substudy B

Inclusion criteria were:

- Male or female participants 18 years and older.
- Willing and able to comply with all scheduled visits, vaccination plan, laboratory tests, lifestyle considerations, and other study procedures.
- Healthy as determined by medical history, physical examination (if required), and clinical judgment of the investigator (healthy participants with preexisting stable disease, defined as disease not requiring significant change in therapy or hospitalization for worsening disease during the 6 weeks before enrollment could be included).
- For participants 65 through 85 years of age, receipt of licensed influenza vaccination for the 2021/2022 Northern Hemisphere season >4 months before study vaccine administration.
- Capable of giving signed informed consent.

Exclusion criteria were:

- Medical conditions
  - Other medical or psychiatric condition including recent (within the past year) or active suicidal ideation/behavior or laboratory abnormality that may increase the risk of study participation or make the participant inappropriate for the study.
  - History of severe adverse reaction associated with a vaccine and/or severe allergic reaction (eg, anaphylaxis) to any component of the study intervention(s).
  - Immunocompromised individuals with known or suspected immunodeficiency, as determined by history and/or laboratory/physical examination.
  - Bleeding diathesis or condition associated with prolonged bleeding that would contraindicate intramuscular injection.
  - Pregnant or breastfeeding.
  - Allergy to egg proteins (egg or egg products) or chicken proteins.
- Prior/concomitant therapy
  - Having significant exposure (someone who was within 6 feet of an infected person for a cumulative total of 15 minutes or more over a 24-hour period) to someone with laboratory-confirmed SARS-CoV-2 infection, COVID-19, or influenza in the past 14 days known before the first study visit.
  - Having SARS-CoV-2 RT-PCR or antigen test in the past 10 days before the first study visit that has not been confirmed as negative.
  - Receiving treatment with radiotherapy or immunosuppressive therapy, including cytotoxic agents or systemic corticosteroids (if systemic corticosteroids are administered for ≥14 days at a dose of ≥20 mg/day of prednisone or equivalent), eg, for cancer or an autoimmune disease,

or planned receipt throughout the study (inhaled/nebulized, intra-articular, intrabursal, or topical [skin or eyes] corticosteroids were permitted).

  - Receipt of blood/plasma products, immunoglobulin, or monoclonal antibodies, from 60 days before study intervention administration, or planned receipt throughout the study.
  - Received or planned to receive a modRNA-platform SARS-CoV-2 vaccine within 28 days of study vaccination.
  - Vaccination with any licensed influenza vaccination for the 2022/2023 Northern Hemisphere influenza season.

- Other exclusions
  - Participation in other studies involving study intervention within 28 days before study entry and/or during study participation.
  - Investigator site staff or sponsor employees directly involved in the conduct of the study, site staff otherwise supervised by the investigator, and their respective family members.
  - Participation in strenuous or endurance exercise through the study.
  - History of heart disease.
  - Screening 12-lead ECG that was consistent with probable or possible myocarditis or pericarditis, or demonstrated clinically relevant abnormalities that may have affected participant safety or interpretation of study results (eg, QTcF interval >450 msec, complete left bundle branch block, signs of an acute or indeterminate-age myocardial infarction, ST-T interval changes suggestive of myocardial ischemia, second- or third-degree AV block, or serious bradyarrhythmias or tachyarrhythmias).

List of principal investigators

Name	Institution	Location
Agard, Malisa	Conquest Research	Winter Park, FL, USA
	Premier Cardiology and Vascular Associates	Maitland, FL, USA
Alvarez-Moreno, Jorge	Millennium Clinical Research	Miami, FL, USA
Anderson, Duane	Excel Clinical Research, LLC	Las Vegas, NV, USA
	Sanjay Vohra, MD, F.A.C.C.	Henderson, NV, USA
Branche, Angela	University of Rochester Medical Center	Rochester, NY, USA
Cabrera, Luis (previously Garcia, Lazaro Miguel)	LMG Research	Miami, FL, USA
Caldwell, Matthew (previously Hendrix, Ernest)	North Alabama Research Center	Athens, AL, USA
	The Heart Center	
Chalhoub, Fadi	Clinical Neuroscience Solutions, Inc. dba CNS Healthcare	Jacksonville, FL, USA
	First Coast Cardiovascular Institute	
Christensen, Tom	Main Street Physician's Care	Little River, SC, USA
Desai, Nitin (previously Oppong, Yaa)	Carolina Institute for Clinical Research	Fayetteville, NC, USA

Dever, Michael	Central Florida Cardiology Group<br>Clinical Neuroscience Solutions, Inc.	Orlando, FL, USA
Doust, Matthew	Arizona Heart Rhythm Center<br>Hope Research Institute<br>The Pain Center of Arizona	Phoenix, AZ, USA
Egelhof, Richard	Alliance for Multispecialty Research, LLC<br>Heartland Cardiology, LLC	Wichita, KS, USA
Essink, Brandon	Meridian Clinical Research<br>Pioneer Heart Institute	Omaha, NE, USA<br>Lincoln, NE, USA
Fink, Ezekiel (previously Rezazadeh, Bahareh)	Cedar Health Research<br>Harmony Heart Group	Irving, TX, USA<br>Plano, TX, USA
Fitz-Patrick, David	East-West Medical Research Institute	Honolulu, HI, USA
Garcia, Lazaro	Entrust Clinical Research	Miami, FL, USA
Geller, Steven	Associates of Cardiology	Silver Spring, MD, USA
Gonzalez Rojas, Yaneicy	Optimus U Corporation	Miami, FL, USA
Guduri, Sridhar (previously Arora, Samir)	Aventiv Research Inc<br>Columbus Cardiovascular Associates, Inc	Columbus, OH, USA
Haffizulla, Jason	Precision Clinical Research	Sunrise, FL, USA
Harper, Charles	Meridian Clinical Research, LLC	Norfolk, NE, USA
Hassman, Michael	Hassman Research Institute<br>Pennsylvania Heart and Vascular Group	Berlin, NJ, USA<br>Jenkintown, PA, USA
Hernandez Rosado, Luis	Innovation Medical Research Center<br>Jackson Medical Group Cardiac Care	Palmetto Bay, FL, USA<br>Miami, FL, USA
Igbinadolor, Awawu	Monroe Biomedical Research	Monroe, NC, USA
Irfan, Muhammad	DM Clinical Research<br>NorthWest Heart Center	Houston, TX, USA<br>Tomball, TX, USA
Kotzker, Wayne	Elixia Infectious Disease, LLC<br>NYU Langone Cardiology Associates	Hollywood, FL, USA<br>Delray Beach, FL, USA
Kutner, Mark	Suncoast Research Group	Miami, FL, USA
Ledo-Sanchez, Gustavo	Best Quality Research, Inc.	Hialeah, FL, USA
Lucasti, Christopher	Penn Medicine<br>South Jersey Infectious Disease	Somers Point, NJ, USA
Mandayam, Sreedhar	Prolato Clinical Research Center	Houston, TX, USA
Martin, Earl	DM Clinical Research<br>Northwest Heart Center	Tomball, TX, USA
Mascolo, Maria	IACT Health	Rincon, GA, USA
Mishkin, David	Proactive Clinical Research, LLC	Fort Lauderdale, FL, USA
Mulligan, Mark	NYU Langone - Center for the Prevention of Cardiovascular Disease NYU Langone Health	New York, NY, USA
Murcia, Alvaro	DBC Research USA	Pembroke Pines, FL, USA
Musante, David	M3-Emerging Medical Research, LLC	Durham, NC, USA
Navarro, Jesus	Genesis Clinical Research, LLC	Tampa, FL, USA

Neutel, Joel	Orange County Heart Institute	Orange, CA, USA
Petersen, Dena	Noble Clinical Research	Tucson, AZ, USA
Philander, Peter	WR-CRCN, LLC	Las Vegas, NV, USA
Poling, Terry	Alliance for Multispecialty Research, LLC<br>Heartland Cardiology, LLC	Wichita, KS, USA
Puente, Orlando	Dr Gerardo A. Polanco, MD<br>Miami Dade Medical Research Institute, LLC	Miami, FL, USA
Ramesh, Mayur	Henry Ford Hospital	Detroit, MI, USA
Ramstad, David	Centricity Research Suffolk Primary Care	Suffolk, VA, USA
Rosen, Jeffrey	Alliance for Multispecialty Research, LLC<br>My Cardiologist	Coral Gables, FL, USA<br>South Miami, FL, USA
Salata, Robert	University Hospitals Cleveland Medical Center	Cleveland, OH, USA
Sanchez, Manuel	Direct Helpers Research Center	Hialeah, FL, USA
Santiago, Fredric	DM Clinical Research	Humble, TX, USA
Sotolongo, Roberto	Research Institute of South Florida	Miami, FL, USA
Surber, Joseph	IACT Health	Columbus, GA, USA
Thakkar, Harish	Mt Olympus Medical Research	Sugar Land, TX, USA
Thomas, Stephen	SUNY Upstate Medical University Global Health Research Unit	Syracuse, NY, USA
Vu, Jack (previously Lavery, Sarah)	Artemis Institute for Clinical Research<br>Inland Valley Cardiovascular Center	Riverside, CA, USA<br>Murrieta, CA, USA
Walsh, Edward	Rochester General Hospital	Rochester, NY, USA
Walters, Alton	Las Vegas Clinical Trials	Las Vegas, NV, USA
White, Alexander	Halifax Health Medical Center<br>Progressive Medical Research	Daytona Beach, FL, USA<br>Port Orange, FL, USA
Ylisastigui, Pedro	Alliance for Multispecialty Research, LLC<br>Millennium Physician Group<br>Robert B. Pritt, DO	Fort Myers, FL, USA
