# Supplementary material for: A Phase 1/2 Randomized Study to Evaluate the Safety, Tolerability, and Immunogenicity of Nucleoside-Modified Messenger RNA Influenza Vaccines in Healthy Adults
Source: Vaccines (Basel). 2025 Apr 3;13(4):383. doi: 10.3390/vaccines13040383 (PMC12031420; doi:10.3390/vaccines13040383)
Supplement: Supplementary file 1 [file vaccines-13-00383-s001.zip › Branche_Table S1.pdf]

Table S1. Strain selections

Vaccine	Strain	
	Substudy A	Substudy B
mIRV	▪ A/Wisconsin/588/2019 (H1N1)<br>*or*<br>▪ B/Phuket/3073/2013 (B/Yamagata lineage)	
bIRV	▪ A/Wisconsin/588/2019 (H1N1)<br>*and*<br>▪ B/Phuket/3073/2013 (B/Yamagata lineage)	
qIRV	▪ A/Wisconsin/588/2019 (H1N1),<br>▪ A/Cambodia/e0826360/2020 (H3N2),<br>▪ B/Phuket/3073/2013 (Yamagata lineage),<br>*and*<br>▪ B/Washington/02/2019 (Victoria lineage)	▪ A/Wisconsin/588/2019 (A/H1N1),<br>▪ A/Cambodia/e0826360/2020 (A/H3N2),<br>▪ B/Phuket/3073/2013 (B/Yamagata lineage),<br>*and*<br>▪ B/Washington/02/2019 (B/Victoria lineage)
QIV	▪ A/Victoria/2570/2019 (H1N1),<br>▪ A/Cambodia/e0826360/2020 (H3N2),<br>▪ B/Washington/02/2019 (B/Victoria lineage),<br>*and*<br>▪ B/Phuket/3073/2013 (B/Yamagata lineage)	▪ A/Victoria/2570/2019 (H1N1),<br>▪ A/Cambodia/e0826360/2020 (H3N2),<br>▪ B/Washington/02/2019-like (B/Victoria lineage),<br>*and*<br>▪ B/Phuket/3073/2013-like (B/Yamagata lineage)

bIRV, bivalent influenza modRNA vaccine; modRNA, nucleoside-modified messenger RNA; mIRV, monovalent influenza modRNA vaccine; qIRV, quadrivalent influenza mod RNA vaccine; QIV, quadrivalent influenza vaccine.
