# Supplementary material for: A Phase 1/2 Randomized Study to Evaluate the Safety, Tolerability, and Immunogenicity of Nucleoside-Modified Messenger RNA Influenza Vaccines in Healthy Adults
Source: Vaccines (Basel). 2025 Apr 3;13(4):383. doi: 10.3390/vaccines13040383 (PMC12031420; doi:10.3390/vaccines13040383)
Supplement: Supplementary file 1 [file vaccines-13-00383-s001.zip › Branche_Table S2.pdf]

Table S2. Grading scale for local reactions and systemic events

	Mild	Moderate	Severe	Grade 4
**Local reaction**				
Injection site pain	Does not interfere with activity	Interferes with activity	Prevents daily activity	ED visit or hospitalization for severe pain at the injection site
Redness	>2.0–5.0 cm	>5.0–10.0 cm	>10 cm	Necrosis or exfoliative dermatitis
Swelling	>2.0–5.0 cm	>5.0–10.0 cm	>10 cm	Necrosis
**Systemic event**				
Vomiting	1–2 times in 24 hours	>2 times in 24 hours	Requires IV hydration	ED visit or hospitalization for hypotensive shock
Diarrhea	2–3 loose stools in 24 hours	4–5 loose stools in 24 hours	≥6 loose stools in 24 hours	ED visit or hospitalization for severe diarrhea
Headache	Does not interfere with activity	Some interference with activity	Prevents daily routine activity	ED visit or hospitalization for severe headache
Fatigue/tiredness	Does not interfere with activity	Some interference with activity	Prevents daily routine activity	ED visit or hospitalization for severe fatigue
Chills	Does not interfere with activity	Some interference with activity	Prevents daily routine activity	ED visit or hospitalization for severe chills
New or worsened muscle pain	Does not interfere with activity	Some interference with activity	Prevents daily routine activity	ED visit or hospitalization for severe new or worsened muscle pain
New or worsened joint pain	Does not interfere with activity	Some interference with activity	Prevents daily routine activity	ED visit or hospitalization for new or worsened severe joint pain

ED, emergency department; IV, intravenous.
