# Supplementary material for: A Phase 1/2 Randomized Study to Evaluate the Safety, Tolerability, and Immunogenicity of Nucleoside-Modified Messenger RNA Influenza Vaccines in Healthy Adults
Source: Vaccines (Basel). 2025 Apr 3;13(4):383. doi: 10.3390/vaccines13040383 (PMC12031420; doi:10.3390/vaccines13040383)
Supplement: Supplementary file 1 [file vaccines-13-00383-s001.zip › Branche_Table S3.pdf]

**Table S3. Participant disposition in substudy A**

| Characteristic | mIRV-A | | | | mIRV-B | | | | bIRV-A/B | | | | qIRV | QIV |
|---|---|---|---|---|---|---|---|---|---|---|---|---|---|---|
| | 3.75 μg | 7.5 μg | 15 μg | 30 μg | 3.75 μg | 7.5 μg | 15 μg | 30 μg | 3.75/ 15 μg | 7.5/ 7.5 μg | 7.5/ 15 μg | 15/ 15 μg | 30 μg | |
| Randomized | 15 | 15 | 15 | 15 | 15 | 15 | 16 | 15 | 15 | 15 | 15 | 15 | 15 | 60 |
| Not vaccinated | 0 | 0 | 0 | 1 | 0 | 0 | 0 | 1 | 0 | 0 | 0 | 0 | 0 | 0 |
| Received vaccination 1 | 15 | 15 | 15 | 14 | 15 | 15 | 16 | 14 | 15 | 15 | 15 | 15 | 15 | 60 |
| Received vaccination 2 | 6* | 10* | 9* | 12* | 6* | 7* | 6* | 2* | 6† | 11† | 6† | 9† | 7‡ | 14§ |
| Discontinued after vaccination 1, did not receive vaccination 2 but continued in the study for safety follow-up | 0 | 0 | 0 | 0 | 0 | 0 | 0 | 0 | 0 | 1 | 1 | 0 | 0 | 2 |
| Discontinued after vaccination 2, but continued in the study for | 0 | 0 | 0 | 0 | 0 | 0 | 0 | 0 | 0 | 0 | 0 | 0 | 0 | 2 |

| | | | | | | | | | | | | | | |
|---|---|---|---|---|---|---|---|---|---|---|---|---|---|---|
| safety follow-up | | | | | | | | | | | | | | |
| Withdrawal after vaccination 1 or 2 from the study | 0 | 0 | 0 | 0 | 1 | 0 | 0 | 0 | 0 | 0 | 0 | 1 | 2 | 0 |

bIRV-A+B, bivalent influenza modRNA vaccine containing 1 A and 1 B strain antigen; modRNA, nucleoside-modified messenger RNA; mIRV-A, monovalent influenza modRNA vaccine containing 1 A strain antigen; mIRV-B, monovalent influenza modRNA vaccine containing 1 B strain antigen; qIRV, quadrivalent influenza modRNA vaccine; QIV, quadrivalent influenza vaccine.

*Participants who were randomized to either mIRV-A or -B and received QIV at vaccination 2.

†Participants who were randomized to bIRV and received QIV at vaccination 2.

‡Participants who were randomized to qIRV and received QIV at vaccination 2.

§Participants who were randomized to QIV and received either mIRV or bIRV at vaccination 2.
