# Supplementary material for: A Phase 1/2 Randomized Study to Evaluate the Safety, Tolerability, and Immunogenicity of Nucleoside-Modified Messenger RNA Influenza Vaccines in Healthy Adults
Source: Vaccines (Basel). 2025 Apr 3;13(4):383. doi: 10.3390/vaccines13040383 (PMC12031420; doi:10.3390/vaccines13040383)
Supplement: Supplementary file 1 [file vaccines-13-00383-s001.zip › Branche_Table S4.pdf]

Table S4. Participant demographics in substudy A

Characteristic	mIRV-A				mIRV-B				bIRV-A/B					
	3.75 µg	7.5 µg	15 µg	30 µg	3.75 µg	7.5 µg	15 µg	30 µg	3.75/ 15 µg	7.5/ 7.5 µg	7.5/ 15 µg	15/ 15 µg	qIRV 30 µg	QIV*
N	16	15	14	14	17	14	15	14	14	16	15	15	15	60
Sex, *n* (%)														
Male	8 (50.0)	8 (53.3)	7 (50.0)	10 (71.4)	10 (58.8)	3 (21.4)	5 (33.3)	5 (35.7)	7 (50.0)	9 (56.3)	3 (20.0)	9 (60.0)	5 (33.3)	28 (46.7)
Female	8 (50.0)	7 (46.7)	7 (50.0)	4 (28.6)	7 (41.2)	11 (78.6)	10 (66.7)	9 (64.3)	7 (50.0)	7 (43.8)	12 (80.0)	6 (40.0)	10 (66.7)	32 (53.3)
Age at vaccination, mean (SD), years	70.3 (3.0)	69.9 (4.7)	69.8 (4.3)	69.2 (2.9)	71.1 (4.9)	70.2 (4.8)	71.4 (4.9)	69.6 (3.7)	68.6 (3.5)	70.4 (4.3)	71.6 (4.8)	71.7 (4.2)	69.9 (2.6)	70.6 (4.7)
Race, *n* (%)														
White	14 (87.5)	14 (93.3)	14 (100)	13 (92.9)	13 (76.5)	13 (92.9)	12 (80.0)	14 (100)	13 (92.9)	15 (93.8)	14 (93.3)	15 (100)	13 (86.7)	52 (86.7)
Black	0	1 (6.7)	0	1 (7.1)	3 (17.6)	1 (7.1)	3 (20.0)	0	1 (7.1)	1 (6.3)	1 (6.7)	0	2 (13.3)	6 (10.0)
Asian	1 (6.3)	0	0	0	1 (5.9)	0	0	0	0	0	0	0	0	2 (3.3)
Not reported	1 (6.3)	0	0	0	0	0	0	0	0	0	0	0	0	0
Ethnicity, *n* (%)														
Hispanic/ Latino	2 (12.5)	1 (6.7)	1 (7.1)	1 (7.1)	7 (41.2)	3 (21.4)	5 (33.3)	5 (35.7)	8 (57.1)	5 (31.3)	4 (26.7)	4 (26.7)	4 (26.7)	13 (21.7)
Non-Hispanic/ non-Latino	13 (81.3)	14 (93.3)	13 (92.9)	13 (92.9)	10 (58.8)	11 (78.6)	10 (66.7)	9 (64.3)	6 (42.9)	11 (68.8)	11 (73.3)	11 (73.3)	11 (73.3)	45 (75.0)
Not reported	0	0	0	0	0	0	0	0	0	0	0	0	0	2 (3.3)

bIRV-A+B, bivalent influenza modRNA vaccine containing 1 A and 1 B strain antigen; modRNA, nucleoside-modified messenger RNA; mIRV-A, monovalent influenza modRNA vaccine containing 1 A strain antigen; mIRV-B, monovalent influenza modRNA vaccine containing 1 B strain antigen; qIRV, quadrivalent influenza modRNA vaccine; QIV, quadrivalent influenza vaccine.

Data are for the vaccination 1 safety population of 65- through 85-year-old participants who received vaccination 1.

*Includes all participants in study receiving licensed QIV at vaccination 1.
