# Supplementary material for: A Phase 1/2 Randomized Study to Evaluate the Safety, Tolerability, and Immunogenicity of Nucleoside-Modified Messenger RNA Influenza Vaccines in Healthy Adults
Source: Vaccines (Basel). 2025 Apr 3;13(4):383. doi: 10.3390/vaccines13040383 (PMC12031420; doi:10.3390/vaccines13040383)
Supplement: Supplementary file 1 [file vaccines-13-00383-s001.zip › Branche_Table S5.pdf]

Table S5. Summary of adverse events in substudy A

AE type, n (%)	mIRV-A 3.75 µg	mIRV-A 7.5 µg	mIRV-A 15 µg	mIRV-A 30 µg	mIRV-B 3.75 µg	mIRV-B 7.5 µg	mIRV-B 15 µg	mIRV-B 30 µg	bIRV-A/B 3.75/ 15 µg	bIRV-A/B 7.5/ 7.5 µg	bIRV-A/B 7.5/ 15 µg	bIRV-A/B 15/ 15 µg	qIRV 30 µg	QIV
N	16	15	14	14	17	14	15	14	14	16	15	15	15	60
Any event	2 (12.5)	3 (20.0)	1 (7.1)	2 (14.3)	3 (17.6)	4 (28.6)	2 (13.3)	3 (21.4)	7 (50.0)	3 (18.8)	6 (40.0)	2 (13.3)	3 (20.0)	9 (15.0)
Related*	0	1 (6.7)	0	0	0	0	0	1 (7.1)	0	1 (6.3)	2 (13.3)	0	2 (13.3)	1 (1.7)
Severe	0	0	1 (7.1)	0	0	0	0	0	0	0	0	0	0	0
Any SAE	0	0	0	0	1 (5.9)	0	0	0	1 (7.1)	0	0	0	0	0
Any AE leading to withdrawal	0	0	0	0	0	0	0	0	0	0	0	0	0	0
Death	0	0	0	0	0	0	0	0	0	0	0	0	0	0

AE, adverse event; bIRV-A+B, bivalent influenza modRNA vaccine containing 1 A and 1 B strain antigen; modRNA, nucleoside-modified messenger RNA; mIRV-A, monovalent influenza modRNA vaccine containing 1 A strain antigen; mIRV-B, monovalent influenza modRNA vaccine containing 1 B strain antigen; qIRV, quadrivalent influenza modRNA vaccine; QIV, quadrivalent influenza vaccine; SAE, serious adverse event.

Data are for the vaccination 1 safety population of 65- through 85-year-old participants who received vaccination 1.

*Assessed by the investigator as related to investigational product.
