# Supplementary material for: A Phase 1/2 Randomized Study to Evaluate the Safety, Tolerability, and Immunogenicity of Nucleoside-Modified Messenger RNA Influenza Vaccines in Healthy Adults
Source: Vaccines (Basel). 2025 Apr 3;13(4):383. doi: 10.3390/vaccines13040383 (PMC12031420; doi:10.3390/vaccines13040383)
Supplement: Supplementary file 1 [file vaccines-13-00383-s001.zip › Branche_Table S6.pdf]

Table S6. Summary of solicited local reactions and systemic events and unsolicited adverse events in substudy B

Type of event, n (%)*	65–85 years of age			18–64 years of age	
Local reaction, systemic event, or adverse event preferred term	qIRV 30 µg	qIRV 60 µg	Licensed QIV	qIRV 30 µg	qIRV 60 µg
	N=115	N=117	N=114	N=131	N=131
Any local reaction†	56 (48.7)	65 (55.6)	35 (30.7)	81 (61.8)	83 (63.4)
Severe local reaction	1 (0.9)	1 (0.9)	0	1 (0.8)	0
Swelling	1 (0.9)	0	0	0	0
Pain at the injection site	0	1 (0.9)	0	1 (0.8)	0
Any systemic event†	51 (44.3)	63 (53.8)	34 (29.8)	75 (57.3)	79 (60.3)
Severe systemic event	1 (0.9)	1 (0.9)	1 (0.9)	3 (2.3)	4 (3.1)
Fever‡	1 (0.9)	1 (0.9)	0	0	2 (1.5)
Fatigue	0	1 (0.9)	1 (0.9)	2 (1.5)	3 (2.3)
Headache	1 (0.9)	0	0	0	0
Chills	0	1 (0.9)	0	0	1 (0.8)
New/worsened muscle pain	0	0	1 (0.9)	1 (0.8)	1 (0.8)
New/worsened joint pain	0	0	0	1 (0.8)	0
	N=116	N=117	N=115	N=131	N=131
Any adverse event§	8 (6.9)	10 (8.5)	8 (7.0)	2 (1.5)	4 (3.1)
Severe adverse event	0	1 (0.9)	0	0	1 (0.8)
Related adverse event	2 (1.7)	2 (1.7)	2 (1.7)	0	0
Injection site pruritis	1 (0.9)	0	0	0	0
Subcutaneous hemorrhage	1 (0.9)	0	0	0	0
Myocarditis	0	1 (0.9)	0	0	0
Pericarditis	0	1 (0.9)	0	0	0
Tinnitus	0	1 (0.9)	0	0	0
ECG T wave inversion	0	0	1 (0.9)	0	0
Troponin abnormal	0	1 (0.9)	0	0	0
Dizziness	0	0	1 (0.9)	0	0
Any serious adverse event∣	1 (0.9)	0	0	0	2 (1.5)
Osteoarthritis	1 (0.9)	0	0	0	0
Pneumonia	0	0	0	0	1 (0.8)
Sepsis	0	0	0	0	1 (0.8)
Viral infection	0	0	0	0	1 (0.8)
Hip fracture	0	0	0	0	1 (0.8)
Related serious adverse event	0	0	0	0	0

ECG, electrocardiogram; qIRV, quadrivalent influenza modRNA vaccine; QIV, quadrivalent influenza vaccine.
The severity scale for local reactions and systemic events is in **Table S2**.
*Participants may have reported ≥1 event.
†Local reactions and systemic events are reported through 7 days after vaccination.
‡Fever >38.9°C to 40°C (there were no fevers >40°C).
§Adverse events are reported through 4 weeks after vaccination.
∣Serious adverse events through 6 months after vaccination.
